# Long-Term Nutrient Cycle in Improved Grain Yield of Dryland Winter Wheat (*Triticum aestivum* L.) under Hydrological Process of Plant Ecosystem Distribution in the Loess Plateau of China

**DOI:** 10.3390/plants12122369

**Published:** 2023-06-19

**Authors:** Hafeez Noor, Anis Ali Shah, Pengcheng Ding, Aixia Ren, Min Sun, Zhiqiang Gao

**Affiliations:** 1 College of Agriculture, Shanxi Agriculture University, Taigu 030801, China; 2 Key Laboratory of Functional Agriculture, Ministry of Agriculture and Rural Affairs, Taigu 030801, China; 3 Collaborative Innovation Center for High-Quality and Efficient Production of Characteristic Crops on the Loess Plateau Jointly Built by Provinces and Ministries, Taigu 030801, China; 4 Department of Botany, Division of Science and Technology, University of Education, Lahore 54770, Pakistan

**Keywords:** nitrogen fertilizers, precipitation, summer fallow, winter wheat, grain yield

## Abstract

Precipitation is the major cause of crop yield variation in rainfed agriculture production in the Loess Plateau. As over fertilization is economically and environmentally undesirable, and crop yield and the resulting returns for N input are uncertain when rainfall variability is high, optimizing N management according to precipitation during fallow season is vital for efficient crop water use and high yield in dryland rainfed farming systems. Results show that the nitrogen treatment rate of 180 treatment significantly increased the tiller percentage rate, and the leaf area index at anthesis, the jointing anthesis, anthesis maturity dry matter, and nitrogen accumulation was closely related to yield. N150 treatment compared to N180 treatment significantly increased the percentage of ear-bearing tiller by 7%, dry substance accretion from jointing to anthesis by 9%, and yield by 17% and 15%, respectively. Our study has important implications for the assessment of the effects of fallow precipitation, as well as for the sustainable development of dryland agriculture in the Loess Plateau. Our results indicate that adjusting N fertilizer inputs based on summer rainfall variation could enhance wheat yield in rainfed farming systems.

## 1. Introduction

Wheat (*Triticum aestivum* L.) is a main source of food globally main, serving about 30% of the world’s population overall [1]. A survey was conducted on farmers regard g Weibei drought sources in the Loess Plateau, and it was found that the amount of nitrogen (N) and phosphate (P) fertilizer used by farmers in dryland areas in the Loess Plateau was generally excessive, accounting for 89% and 75%, respectively, and the average amount applied was N180 kg ha^−1^ and N210 kg ha^−1^, respectively. The amount of nitrogen fertilizer and phosphate (P) fertilizer used by farmers was much higher than that of potassium (K) fertilizer. This is mainly due to the fact that more than 70% of farmers in the dryland wheat area do not apply potash fertilizer. Farmers’ unreasonable fertilization causes soil nutrient imbalance [2]. The yield of dryland wheat in the Loess Plateau is low and unstable, but the N application is not different from that in other areas, and fertilizer utilization efficiency is low [2,3]. The nitrogen partial productivity of farmers applying nitrogen in southern Shanxi could only reach 180 kg kg^−1^ [4,5]. The agronomic efficiency of N fertilizer, P fertilizer, and K fertilizer in the dry wheat area in the Loess Plateau reached 6.4, 7.1, and 7.1 kg ha^−1^, respectively, and the agronomic efficiency of N fertilizer, P fertilizer, and K fertilizer increased to 8.3, 8.3, and 10.0 kg ha^−1^, respectively, after the optimization of fertilization, significantly improving fertilizer efficiency [6]. Among all the countries in the world, China is one of the top wheat producers in the world. The North China Plain (NCP) is one of the most important wheat production areas in China, with 25% of nationwide food production [7,8]. N is a main important to crop manufacture, as it directly impacts dry-matter manufacture of crop plants by prompting the leaf area, radiation interruption, and N use productivity [8,9,10]. In numerous parts of the world, a great increase in N fertilizer input is essential to increase crop profits [9]. However, the rise in crop profits has not coordinated an increase in N fertilizer contribution. For example, from 1980 (9.3 Mt) to 2012 (24 Mt), the rise in N fertilizer effort was 158%, which was related to a 70% increase in China’s crop yield [11].

Additionally, excessive N input is also an indication of N fertilizer excess, which causes various environmental difficulties, such as soil acidification, N_2_O releases, and reduced soil microbial movement [8,12]. Natural precipitation is the only water basis for wheat growth in dryland, which determines the soil water status. Soil water shortfall affects the growth and development of wheat in each key growth stage, negatively affects water use of wheat and plant dry-matter accumulation and transport, and leads to a decline in wheat yield and grain quality [12]. Therefore, the serious growth period of wheat and the shortage of water in the critical growth period of wheat is usually caused by a deficiency of soil water standby due to inadequate precipitation in the early stage or excessive consumption of soil water, resulting in a decline in wheat yield [13].

Precipitation during the fallow period in dryland wheat fields plays an important role in promoting tillering, regreening jointing, and establishing excellent populations and healthy individuals in dryland wheat fields [14]. Wheat yield in dryland had a significant positive linear correlation with precipitation during the fallow period (*p* < 0.05) but had no significant association with precipitation in the growing season. N is an important nutrient for crop growth. Rational application of N fertilizer not only supplements soil nutrients, but also promotes soil water N interaction and promotes crop growth [15]. The N application reduced soil water consumption before jointing in dryland wheat, provided sufficient soil water for later growth and development of wheat, improved water-use efficiency and spike number per area, and thus increased yield. Soil water intake in dryland wheat-growing season is far greater than precipitation. Long-term excessive nitrogen application causes excessive consumption of soil reservoirs during wheat-growing season, and it is difficult to maintain a stable soil reservoir for a long time, limiting the improvement in water-use efficiency and yield [16].

**Figure 1:**
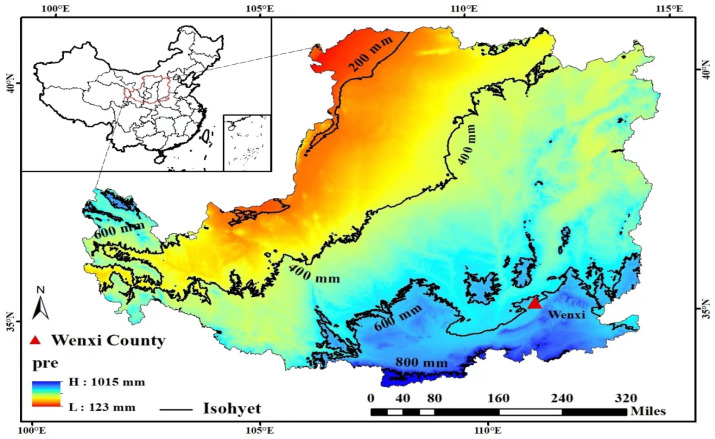
Location of experiment site in the Loess Plateau. The regional distribution of annual precipitation is shown in different colors on the map [13].

Excessive N application would make the soil water consumption of dryland wheat too high before jointing, and the soil water storage in shallow and middle layers was insufficient, which would affect the sowing of wheat after jointing and lead to yield reduction [17]. The relationship between precipitation and yield in the Weibei drought source is shown in Figure 1, and it is also presented that by using rainfall in the fallow period to predict yield and guide nitrogen application could reduce N by N180 kg ha^−1^ and ensure stable yield. Thus, the objectives of this work were as follows: (i) to establish a reference range for summer rainfall describing wheat-cultivation year types based on fallow season precipitation; (ii) to assess the responses N fertilization’s rate of grain yield, spatiotemporal dynamics in soil water storage, use rate in dry land, and winter wheat under varying rainfall conditions; and (iii) to determine the optimal N input rates for each year type to realize maximum grain yield.

## 2. Results

### 2.1. Agronomic Characteristics

The leaf area index (LAI) of wheat at the jointing stage was significantly decreased (by 15.6%) following the N application of N150 treatment compared to N210 treatment. Following an increase in N application rate, LAI did not change significantly during anthesis, which was about 3.39–3.54. The LAI at the jointing stage was significantly decreased (by 20.5%) following the N application of N180 treatment compared to N210 treatment. Increasing the N application rate of N0, the LAI did not show any significant change during the flowering stage, reaching about 3.67–3.86 (Table 1). The N application rate of N150 treatment and N180 treatment in all years were beneficial to increase jointing to flowering LAI of dryland wheat.

#### 2.1.1. Effects of Nitrogen Fertilizer on Plant Dry-Matter Accumulation in Dryland Wheat

The dry-matter accumulation at the jointing stage of dryland wheat was significantly reduced by 12.9% following the N application of N150 treatment compared to N210 treatment. The increase in dry-matter accumulation at anthesis had no significant variation, about 8.1–8.6 kg ha^−1^. Associated with N210, N150 treatment significantly reduced dry-matter accumulation at jointing stage by 12.7%, while with the rise in N application level, dry-matter accumulation at anthesis had no significant variation, which was about 10.1–10.9 kg ha^−1^ (Supplementary Table S1). The N application rate of N150 treatment during the drought year and N180 treatment during the rainfall year was additionally helpful to dry-matter accumulation in dryland.

The dry-matter accumulation of wheat at mature the stage was significantly improved by 10.7% following the N application of N150 treatment compared to N210 treatment. The dryland wheat harvest index increased to 5.5% following N210 treatment compared to N180 treatment, which significantly improved dry matter in dryland at the maturity stage by 9.6% (Supplementary Table S2). N150 significantly increased dry-matter accumulation from jointing to flowering stage in dryland wheat by 14.8% compared to N210 treatment. The dry-matter accumulation of anthesis to the maturity of wheat in dryland was significantly improved by 34.5%. N210 treatment compared to N180 treatment significantly increased dry-matter accumulation from jointing to the flowering stage in dryland by 9%. The dry-matter accumulation of anthesis to the maturity stage was significantly increased by 34.3%.

#### 2.1.2. Effects of Nitrogen Fertilizer on Nitrogen Accumulation at Anthesis and Maturity Stage

The N application rate of N150 was 19.4% less than that of N210 at the jointing stage. Plant N accumulation decreased at the flowering stage by up to 11.7%. N accumulation was significantly reduced at the mature stage by 12.1%. N210 treatment compared to N180 treatment significantly reduced N accumulation at the flowering stage by 10.7%. It also significantly reduced the N accumulation of plants at anthesis and the maturity stage by 6% (Table 2) in N150 treatment and N180 treatment in the same year reduced both N fertilizer and the N buildup of wheat in dryland at different growth stages.

**Figure 2:**
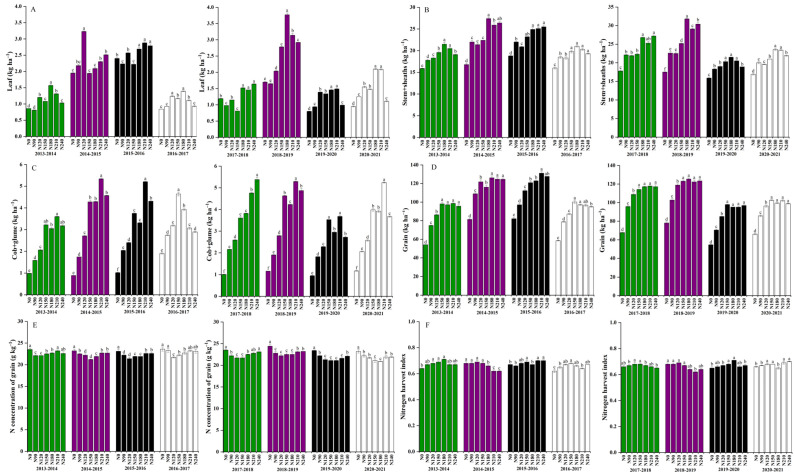
Effects of nitrogen rate on nitrogen accumulation of different organs and N concentration of grain in dryland winter wheat. The means are not significantly different within a given season when followed by the same lowercase letter using LSD at *p* < 0.05. (**A**) leaf, (**B**) Stem + sheath, (**C**) cob + glume, (**D**) Grain (**E**) N concentration of grain (**F**) N harvest index.

The N application rate of N150 treatment significantly reduced leaf N accumulation by 25%, related to N210 (Figure 2). N0 was not significant and was likely a variant of N accumulation of Stem + leaf sheath and spike + glume at maturity, which were 20.2–25.2 and 3.5–4.1 kg ha^−1^, respectively. The N application rate of N210 treatment compared to N180 treatment could increase leaf N accumulation in dryland at maturity by 3%. The accumulation of cob + glume nitrogen was significantly decreased by 24% in dryland at the maturity stage. N210 treatment presented significant variance in N accumulation of stem + leaf sheath at maturity, which was about 23.3–27.7 kg ha^−1^. The N application rate of N210 treatment compared to N180 treatment significantly increased organs and N concentration of grain in dryland by 9%.

#### 2.1.3. Effects of Nitrogen Fertilizer on Yield Component and Protein Content Nitrogen-Use Efficiency

**Figure 3:**
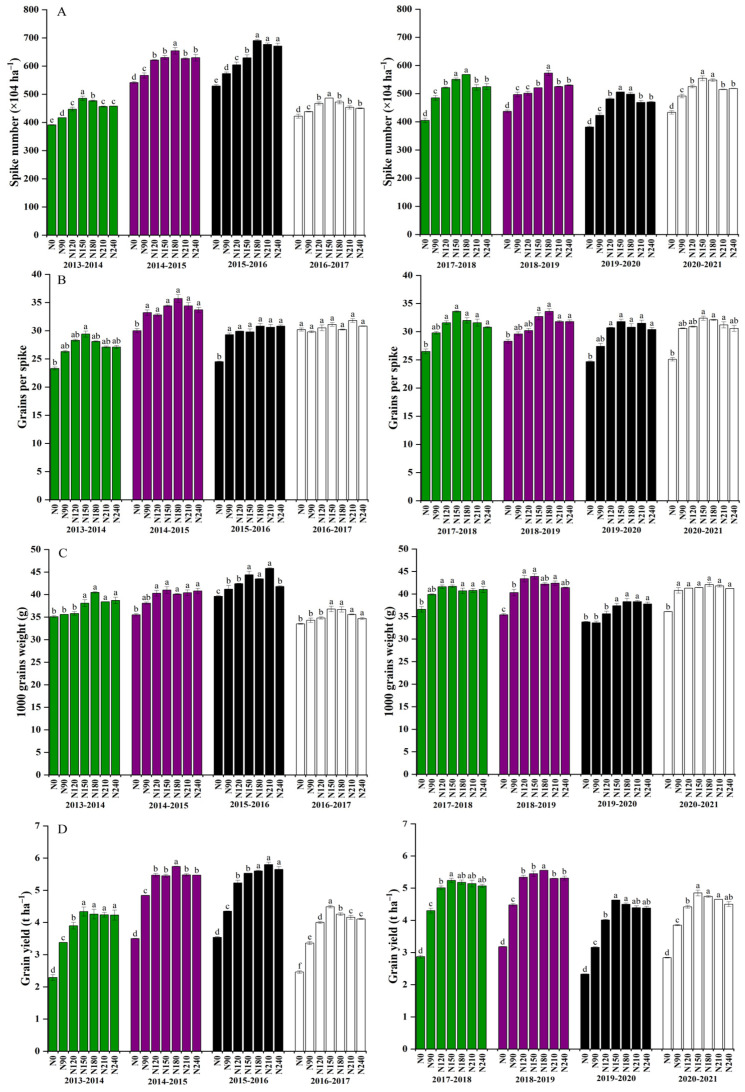
Effects of nitrogen rate on grain yield and components in dryland wheat. (**A**) (spike number), (**B**) (Grain number per spike), (**C**) (1000–grain weight), and (**D**) (highest grain yield). The means are not significantly different within a given season when followed by the same lowercase letter using LSD at *p* < 0.05.

The spike number increased by 7.6% following the N application of N150 treatment compared to N210 treatment (Figure 3A). With the increase in N application rate, grain number per spike and thousand grain weight did not change significantly. The N application rate of N210 compared to spike number increased by 5.3% upon N180 treatment. Proliferation of N application rate, grain number per spike, and thousand grain weight had no significant alteration.

Grain number per spike was the highest following N application of N150 treatment, which was significantly different from N0 treatment (Figure 3B). N180 treatment was the highest, which was significantly different from N0 treatment. The highest N application rate was found during N150 treatment per grain per spike, which was significantly different from N0 treatment.

The 1000–grain weight was the highest with N180 treatment, and the variance between N180 treatments was significant (Figure 3C). The thousand grain weight was the highest with N150, while the thousand grain weight of N210 was the highest, reaching 46.9 g, which was significantly diverse from that of N0, N90, N120, and N240 treatment.

The highest grain yield was 555.6 × 1 kg ha^−1^ (Figure 3D). The highest yield was 660.1 × 106 kg ha^−1^. There was significant variance between N180 treatments of other N application rates from that of N0, N90, N120, N210, and N240 kg ha^−1^ treatment.

**Figure 4:**
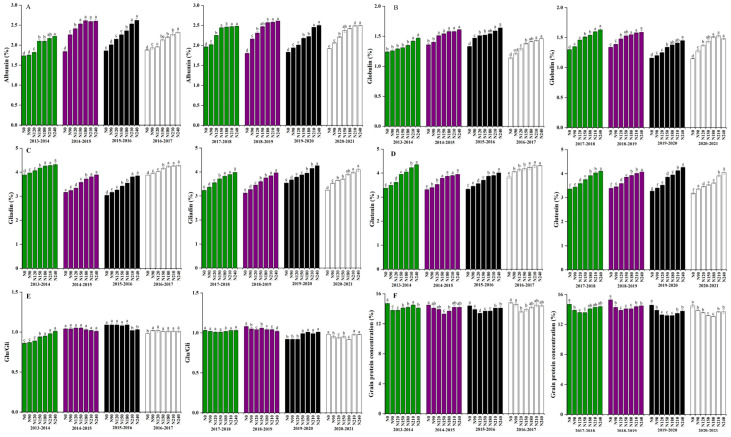
Effects of nitrogen rate on grain protein concentration under different nitrogen rates in dryland wheat. Contents of albumin, globulin, glutenin and glaidin in dryland wheat grains had no significant difference. (**A**) (Albumin), (**B**) (Globulin), (**C**) (Glutenin), (**D**) (Glaidin), (**E**) (Glu/Gli), and (**F**) (Grain protein concentration). The means are not significantly different within a given season when followed by the same lowercase letter using LSD at *p* < 0.05.

The upsurge in N application rate and the contents of albumin, globulin, glutenin and gliadin in dryland wheat grains had no significant differences, which were 2.19–2.34%, 1.37–1.44%, and 3.98–4.17%, respectively (Figure 4A–D). Associated with N210 treatment, N150 treatment significantly reduced grain gliadin content in dryland wheat. With the rise in N application levels, the contents of albumin, globulin, and glutenin in dryland wheat grains had no significant difference, which were 2.42–2.54% and 3.77–3.96%, respectively. The N application rate of N210 treatment compared to N180 treatment significantly reduced grain gliadin content in dryland wheat.

The N application rate of N210 treatment compared to N use efficiency and N partial productivity of dryland wheat were significantly increased by 36% and 32%, respectively. The N application rate of N180 treatment compared to N210 treatment significantly increased N use efficiency and N partial productivity of dryland wheat by 17% and 15%, respectively (Table 3). In conclusion, nitrogen application of N150 treatment in all years significantly improved the nitrogen-use efficiency and nitrogen partial productivity of dryland wheat.

#### 2.1.4. Correlation between Nitrogen Accumulation and Leaf Area Index Yield Component Factors at Different Stages

There was a significant correlation between wheat yield in dryland and N accumulation in sowing to jointing, jointing to flowering, and flowering to mature stages in different rainfall years. The number of spikes was significantly correlated with N accumulation in jointing stage and significantly correlated with the N accumulation in the jointing–flowering stage. The number of grains per spike was significantly correlated with N accumulation from jointing to flowering and flowering to maturity. Grain weight of 1000 was significantly correlated with N accumulation in the jointing and flowering stages and significantly correlated with N accumulation in the flowering and mature stages. Moreover, the yield and component factors were more closely related to the N accumulation of the seeding, jointing, jointing, flowering and flowering, and mature stages (Table 4). In conclusion, wheat yield and component factors were significantly affected by N accumulation in the jointing and flowering stage.

The yield of dryland wheat was significantly correlated with tiller heading rate and the LAI at the jointing and flowering stages in different rainfall years. The number of spikes was significantly correlated with tiller completion rate and the leaf area index at the jointing stage and flowering stage. The number of grains per spike was significantly correlated with the leaf area index at the jointing and flowering stages. The 1000 grain weight was significantly correlated with the leaf area index at the flowering stage. In addition, the yield and component factors were more closely related to the tiller heading rate and leaf area index at the jointing and flowering stages than during the dry years (Table 5). In conclusion, tiller heading rate and the leaf area index of the jointing–flowering population significantly affected the formation of wheat ear number and yield in dryland.

## 3. Discussion

### 3.1. Effects of Nitrogen Fertilizer on Precipitation-Fallow-Use Efficiency in Loess Plateau Dryland Wheat

The synergistic effects of N fertilizer and soil moisture determines wheat yield. Insufficient soil moisture reduces the positive effect of soil N on wheat production, while heavy precipitation or excessive irrigation amount lead to leaching loss of soil nitrogen, which also has an adverse effects on yield [18]. If a soil water deficit appeared at the tillering stage of wheat, it would have a serious effect on spike number. If it appeared in the booting to flowering stage, it would significantly affect the grain number per spike of wheat [19]. If it occurs at the post-flowering grain-filling stage, the 1000 grain weight formation would be significantly affected [20]. Higher soil nitrogen nutrient levels accelerates plant growth, thus depleting soil water reserve. Although wheat can produce more grains, its grains may not be full because of soil water shortage [21,22]. In order to obtain advanced grain yield and crop-water-production proficiency, N fertilizer input must be adjusted according to precipitation. Therefore, scientific fertilization must consider the accumulation and utilization of water. The results showed that in drought years, N210 compared with N150 significantly increased the water-use efficiency of crops by 6.1%. N210 compared to N180 significantly increased the water-use efficiency of crops by 8.5%. This indicates that one of the reasons for high yield in different precipitation year types was higher water-use efficiency [23]. During drought treatment in the wheat-sowing stage and the wintering stage, the plant biomass of wheat showed a significant downward trend, and the wintering stage could not compensate for the biomass loss caused by drought [24]. During the 8 years of experiment in this study, the precipitation-fallow-use efficiency (PFUR) was equal to the fallow soil water storage ratio (SWSR), indicating that the fallow precipitation stored in the soil could only meet a part of the water consumption demand of subsequent crops. The soil water accumulated during the fallow period could meet the water consumption before the jointing stage of dryland wheat. The soil water accumulated during the fallow period can meet the water consumption before anthesis of dryland wheat. This is similar to the conclusion proposed by [25]. Precipitation in the leisure period can affect the jointing stage. Dryland wheat gradually uses soil water in deeper layers with its growth stage [26]. Research on the spatiotemporal dynamics of water in dryland wheat fields in Shanxi shows that a N application level of N150 significantly improved soil water consumption in 80/240 cm soil layers during the jointing to flowering stage by 36.6%, related to that of N210. There was a significant positive parallel between soil water intake and spike number between jointing to flowering (80, 240 cm a = 1.7, R2 = 0.45, *p* < 0.001 compared to N210). The soil water consumption of N180 significantly increased by 22.7% in the 80–240 cm soil layer, and there was a significant positive correlation between the soil water consumption of N180 and the spike number (a = 2.9, R2 = 0.47, *p* < 0.001). The results showed that the N application rate significantly increased the water consumption of jointing to flowering soil in dryland, promoted tiller spike creation, improved the actual spike number, and then increased the yield regardless of drought years or all year round.

### 3.2. Effects of Nitrogen Accumulation in Loess Plateau Dryland Winter Wheat

The process of crop growth, progress, and yield creation is the process of material transformation between crops and the environment, as well as the process of material accumulation and transformation between crop organs [27,28]. For dryland wheat, the period prone to water shortage is the key growth period that limits the yield of dryland wheat [29]. The influence of precipitation at various growth stages on wheat yield in dryland was in the following priority order: the sowing stage and the jointing stage, followed by other growth stages. The main limiting factor of wheat yield in dryland was precipitation upon sowing emergence during the jointing stage. Therefore, both the sowing stage and the jointing stage are key periods affecting the number of spikes in dryland winter wheat [30]. The key growth period of tiller differentiation in dryland wheat is before and after jointing, which also has an important influence on spike number at the mature stage [2,12]. It is believed that ineffective tiller reduces the optimal effective population number of spike wheat, and excessive ineffective tiller would also have a negative influence on the amount of ear and grain-of-weight wheat. As a result, a huge number of studies have focused on delaying the wheat development of wheat-individual-nutrient competition environment and resources, thus affecting the wheat tiller earing rate of late, which has a large influence on yield and its components [2]. In drought years, the sowing percentage of wheat significantly increased by 20.3% with N150 compared to N210. The tiller of dryland wheat was significantly increased by 7% at N180. N150 significantly improved dry-matter accumulation from jointing to flowering in dryland wheat by 14.8% compared with N210 [31]. N210, compared to N180, significantly increased the dry-matter accumulation of jointing to flowering wheat in dryland by 9%. Linking analysis showed that dry-matter accumulation from jointing to flowering was significantly positively correlated with tiller percentage in dryland [4]. A suitable N application rate can significantly increase dry-matter accumulation from the jointing to flowering stage, increase tiller rate, promote the formation of effective tiller, and increase yield. The relationship between sources is the material basis for yield formation. The results show that grain profit decreased with the rise in dry-matter accumulation.

### 3.3. Effects of Nitrogen Fertilizer on Wheat Yield in the Loess Plateau Dryland Wheat

Regarding dryland wheat in the Loess plateau, the thinking of leisure period precipitation is used to determine that the fertilizer rate is not fresh [30]. In Shanxi, the secretary-general of a long-term experimental study showed that the output of rainfed area precipitation effects the fertilizer, especially during leisure period precipitation; regardless of the leisure period precipitation in fertilizer inputs, the result is not ideal [23]. The association between precipitation spreading and dry wheat yield in Changwu for 25 consecutive years showed that yield was significantly positively correlated with precipitation in the fallow period (*p* < 0.05), but it was not significantly correlated with precipitation in the growing period [32]. There was a significant non-linear positive correlation between precipitation in the fallow period yield and the proposed yield prediction model for precipitation in the fallow period to simulate the yield performance of precipitation in different fallow periods to calculate the required N application amount by simulating yield. All of the above studies are good explorations of determining fertilizer input based on rainfall in the fallow period [33]. The distribution features of rain in the fallow period in dryland wheat were examined, and the annual types were divided by rainfall in the fallow period. Furthermore, the correct N application amount for the changed annual types of precipitation in the fallow period was explored by field experiments with altered N gradients. The results showed that in drought years, N150 significantly improved by 5.0% compared to N210, and N240 decreased by 28.6%. Associated with N180, N210 significantly increased wheat yield in dryland by 5.5%, while N210 kg ha^−1^ N fertilizer was reduced by 14.3%. In drought years, the panicle number increased by 7.6% at N150 compared with N210, but there were no significant differences in grain number per spike and 1000 grain weight. N210 compared to N180 significantly increased spike number by 5.3%, but there was no significant difference in grain number per spike and 1000 grain weight [34]. The differences in climatic conditions and soil fertility in different test areas may lead to differences between studies [13]. The reasonable nitrogen application amounts of N150 kg ha^−1^ and N180 were similar to the effects of this study when the annual precipitation was normal (500–600 mm) and humid (600 mm) (Figure 2). This study only takes Wenxi in Shanxi Province as an example. Climate and environmental differences in different regions have a great impact on the research results. In the dry farming wheat region of the Loess Plateau, the annual types are divided according to the precipitation during the fallow period, which can be adjusted based on the local precipitation data in each region to be applicable to the local production reality. In conclusion, the yield increased by 5% and N decreased by 28.6% in drought years with N150. All year round, N180 increased yield by 5.5% and reduced N by 14.3%. The input nitrogen fertilizer directly affects the protein comprised of grains [35]. Meanwhile, the protein content of grains is restricted by the process of N absorption, transport, buildup, and re-transport of wheat plants [36]. The results show that N150 significantly reduced grain protein comprised of dryland wheat by 0.5% compared with N210 in drought years. N210 compared to N180 significantly reduced grain protein of dryland wheat by 0.6%. The results indicate that suitable N application based on fallow precipitation reduced the grain protein content of dryland wheat, and further screening and exploring the potential of N utilization of dryland wheat varieties would be one of the effective directions.

## 4. Materials and Methods

A field experiment was conducted at the Wheat Agriculture Station (35°20′ N, 111°17′ E) in Wenxi county, Shanxi Province, China. The main soil types were loam, as shown in Table 1. According to the classification technique of rain year varieties in the fallow period, precipitation in the fallow period was fewer than 230.7 mm, precipitation in the fallow period was 230.7–439.0 mm, and precipitation in the fallow period is wet. The precipitation during 2013–2014, 2014–2015, 2015–2016, and 2016–2017 was 150.4 mm, 209.5 mm, 131.6 mm, and 154.7 mm, respectively, which were dry years. The precipitation in 2018–2019, 2019–2020, and 2020–2021 were 331.4 mm, 441.1 mm, and 346.4 mm, respectively (Table 6).

### 4.1. Experimental Design

The winter wheat cultivar ‘Yunhan 20,410′ was obtained from the Wenxi Agriculture Bureau, Wenxi, China. A single-feature random block design was used to set the present application of 0, 90, 120, 150, 180, 210, and 240 N kg ha^−1^ of N fertilizer in the study. The plot area was 10 m × 6 m = 60 m^2^. Three repetitions of N210 kg ha^−1^ classified the conventional nitrogen application rate of farmers (the average nitrogen application rate in the survey area was 205 kg ha^−1^), and 0 kg ha^−1^ was set as the individual subplot size, which was 8 m in length and 4 m in width. The previous wheat crop was harvested with 20–30 cm of high stubble, which was carried out in early and middle July, and the land was leveled with shallow rotation at the end of August. The seeds were sown at the end of September or early October with 60 kg P_2_O_5_ ha^−1^ and 30 kg K_2_O ha^−1^ as phosphate fertilizer and potassium fertilizer before sowing, whereas the higher N treatment (N240) was included to ensure maximum grain yield was achieved.

### 4.2. Soil Moisture

At sowing, jointing, anthesis, and maturity, soil samples were collected down to a 3 m depth with a soil drill. Soil water storage (SWS) was calculated as follows [26].

SWS = BD/*ρ*w × SWC × H

where SWS, BD, SWC, H, and *ρ*w represent the soil water storage (mm), bulk density (g cm^–3^), water content (g water g^−1^ dry soil), depth (mm), and water density, respectively. BD and SWC were calculated according to the method of [26].

#### 4.2.1. Soil Organic Carbon

After drying, soil samples were screened at 0.25 mm, and the total soil organic carbon content was determined using the potassium dichromate volumetric method.

#### 4.2.2. Soil-Alkali-Hydrolyzed Nitrogen

After air drying, soil tasters were partitioned using a 1 mm sieve, and the contented of alkali-hydrolyzed nitrogen was determine using the alkali-hydrolyzed diffusion technique. Soil-available p and available K soil samples were dried and screened at 1 mm, and the contents of available P and K were dissolved in 0.5mol L^−1^ NaHCO_3_ extraction molybdenum–antimony-resistance colorimetric technique and 1mol L^−1^ NH_4_OAC extraction-flame spectrophotometry, respectively.

### 4.3. Agronomic Traits and Dry-Matter Accumulation

Population dynamics: Three rows of wheat plants at 0.667 m^2^ were selected at a fixed-point stage of wintering, jointing, flowering, booting, and maturity to investigate population dynamics. The 10 plants were selected from each plot at each growth stage to measure the length, width, and number of green leaves of the two inverted leaves and calculate the leaf area. Leaf area (cm^2^) = length × width × 0.73 × number of green leaves.

### 4.4. Leaf Area Index

According to the method, 10 plants were selected from each plot in each growth stage to measure the width, length, and number of green leaves of the inverted second leaf and calculate the leaf area. Leaf area (cm^2^) = length × width × 0.73 × number in green leaves.

### 4.5. Dry-Matter Quality

Twenty strains of plant growth were taken as representatives and divided into two groups. The wintering phase (the plant); jointing phase (leaf and stem); flowering period (leaf, ear, stem, and leaf sheath); and adulthood (leaf sheath, leaf, stem, cob + glume shell and grain). Each part was placed on an annotation processing Kraft paper bag, first at 105 °C for 1 h, before the oven was adjusted to 70 °C. Then, they were baked to a continuous weight, and the dry-matter weights were weighed and recorded. The samples were weighed and crushed.

### 4.6. Yield and Yield Components

The effective spike number of the 0.667 m^2^ evenly growing wheat-sample segment was investigated in each plot by removing side rows at the maturity stage. Then, 20 spikes were arbitrarily selected from individual plots, and the average grain number per spike was calculated after drying. Five groups of grain samples (1000 grains per group) were randomly selected, and the 1000 grain weight was determined by the models. For yield measurements, 20 m^2^ was randomly selected from each treatment. Grain moisture content was measured using a grain moisture meter (KETT PM-8188-A, dehua road, jiading distinct, shanghai, China) and converted according to the national grain storage standard water content (13%), which was the actual yield.

The accumulation, partitioning, and translocation of dry matter and N were calculated using the following equations [37,38]:

Post-anthesis dry-matter production

(DM_post_, kg ha^−1^) = Total dry weight at maturity − TDW_as_ (1)

Harvest index

(%) = Grain dry weight/Total dry weight at maturity (2)

Post-anthesis accumulated N (N_post_, kg ha^−1^) = TN − TN_as_ (3)

N harvest index (NHI, %) = GN/TN (4)

where TDW_as_ (kg ha^−1^) is the total dry weight at anthesis. TN (kg ha^−1^) and TN_as_ (kg ha^−1^) are the total N quantity at maturity and anthesis, respectively. GN (kg ha^−1^) is grain N content.

### 4.7. Crop Water Productivity and Total Evapotranspiration (ET)

Crop water productivity (kg grain yield ha^−1^ mm^−1^ ET) was calculated as follows:

CWP = Y/ET(5)

where Y is the grain yield (kg ha^−1^) and ET (mm) is the total evapotranspiration during the wheat growth period from sowing to maturity.

Total Evapotranspiration (ET) was calculated using the water balance equation. Thus, there was minimal drainage below the measurement depth during the growing season. Hence, the field water balance equation was simplified to [39]:

ET = *P* + Δ*SWS*_*S*−*M*_ + *IR* − *SR* − *DWP* (6)

where precipitation *P* (mm), Δ*SWS_S−M_* (mm), and *IR* (mm) represented the total rainfall during the growing season, soil water storage (*SWS*) (0–300 cm) change between the start and end of the growing season, and irrigation rate (*IR*), respectively. In the present study, *IR* = 0 because no irrigation was applied at any time during the experiment. *SR* (mm) represented the surface runoff (all plots were arranged on flat farmland surrounded by high ridges that prevented surface runoff), and *DWP* (mm) represented deep-water percolation (*DWP*). In the study region, the soil had a large water-holding capacity.

### 4.8. Nitrogen Content in Plants

The crushed plant samples were boiled with H_2_SO_4_-H_2_O_2_, cooled to a constant volume of 50 mL, and diluted 10 times. Subsequently, 1 mL of diluent was taken, and 1 mL of EDTA-methyl red solution was dropped and titrated with 0.6 N Na OH solution. After the solution turned from red to yellow, 5 mL sodium hypochlorite solution and 5 mL phenol solution, and distilled water added at a constant volume of 50 mL and shaken.

After 1 h, OD_625_ was determined with a colorimeter of a 1 cm light diameter, and the blank dissolving solution with the same dissolving solution and various reagents was zeroed. Different 20 ears of wheat with uniform growth were picked in the sample at the mature stage, and the grains were stripped and placed in the oven to dry at 70 °C. The dried grains were crushed by a disc experimental grinding mill (Perten, Sweden) for the determination of protein content. The nitrogen content of grains was determined using theH_2_SO_4_-H_2_O_2_ indiophenol blue colorimetric method, and the protein content was multiplied by 5.7. Each sample was repeated three times. An incessant extraction technique was used to regulate the protein components of grains.

### 4.9. Statistical Analysis

Experimental statistics were statistically examined using Microsoft Excel 2016 and Statistix 8.0 (Analytical Software, Tallahassee, FL, USA), and the figure was produced using Origin Lab pro 2021b (Origin Lab Corporation, Northamptn, MA, USA). Comparisons among numerous groups were achieved using Tukey’s honestly significant difference (HSD) test. Possibility values *p* < 0.05 considered statistically significant. Statistix 8.0 software was used for alteration analysis.

## 5. Conclusions

The nitrogen application based on fallow precipitation in dryland wheat fields was beneficial to the water use of jointing and flowering soil and the accumulation of plant nitrogen and dry matter in dryland wheat. The dry substance buildup from jointing to flowering was improved by 14.8%, while grain protein content significantly decreased by 0.5%. Furthermore, the soil-water-conservation treatment could not change the fact that the soil water storage at sowing varies with summer rainfall and that “drought at sowing” has a more adverse effect on yield than “drought in the growing season.” Thus, small holders need to adjust N inputs based on the fallow season precipitation to improve yield in rainfed farming systems. It significantly increased tiller percentage by 7% and dry-matter accumulation from jointing to flowering by 9%. Our study has important implications for assessing the effects of fallow precipitation, as well as for the sustainable development of dryland agriculture in the Loess Plateau.

## Figures and Tables

**Table 1:** Effects of nitrogen rate on leaf area index at jointing stage and anthesis stage in dryland wheat.

Year	Jointing Stage (JS)							Anthesis Stage (AS)						
	N0	N90	N120	N150	N180	N210	N240	N0	N90	N120	N150	N180	N210	N240
2013–2014	1.40 ^c,d^	1.50 ^b,c^	1.61 ^b,c^	1.69 ^b^	1.92 ^a^	1.95 ^a^	1.98 ^a^	2.64 ^e^	3.35 ^d^	3.44 ^c^	3.50 ^c^	3.22 ^d^	3.95 ^a^	3.69 ^b^
2014–2015	1.60 ^d^	1.80 ^b,c^	1.85 ^c^	1.83 ^c^	1.94 ^c^	2.23 ^b^	2.37 ^a^	3.15 ^c,d^	3.64 ^b^	3.68 ^a,b^	3.73 ^a,b^	3.86 ^a^	3.85 ^a^	3.86 ^a^
2015–2016	1.95 ^b,c^	1.92 ^b,c^	1.97 ^c^	1.94 ^c^	1.98 ^c^	2.53 ^a^	2.27 ^a^	3.25 ^b^	3.63 ^a,b^	3.66 ^a,b^	3.69 ^a,b^	3.68 ^a,b^	3.79 ^a^	3.72 ^a,b^
2016–2017	1.20 ^f^	1.46 ^e^	1.55 ^d^	1.64 ^d^	1.81 ^c^	1.86 ^b^	1.96 ^a^	2.70 ^c,d^	3.12 ^b,c^	3.16 ^b,c^	3.36 ^b^	3.36 ^b^	3.56 ^a^	3.53 ^a^
2017–2018	1.90 ^b,c^	1.89 ^b,c^	1.92 ^c,d^	2.10 ^b^	2.10 ^b^	2.35 ^a^	2.37 ^a^	3.11 ^d^	3.54 ^c^	3.61 ^b,c^	3.66 ^b,c^	3.78 ^b^	3.91 ^a^	3.88 ^a^
2018–2019	1.89 ^b,c^	1.98 ^b,c^	1.99 ^c,d^	2.07 ^c,d^	2.14 ^c^	2.38 ^b^	2.47 ^a^	3.15 ^d,e^	3.50 ^d^	3.61 ^c,d^	3.70 ^c^	3.86 ^b^	3.99 ^a^	3.87 ^b,c^
2019–2020	1.59 ^d^	1.68 ^b,c^	1.78 ^c^	1.70 ^c,d^	1.99 ^a^	1.84 ^b^	1.92 ^a^	2.90 ^d,e^	3.14 ^d^	3.31 ^c^	3.48 ^b,c^	3.37 ^c^	3.70 ^a^	3.55 ^b^
2020–2021	1.12 ^e^	1.49 ^d^	1.68 ^c^	1.70 ^c,d^	1.89 ^b,c^	2.10 ^a^	1.98 ^a,b^	2.71 ^e,f^	3.15 ^e^	3.24 ^d^	3.33 ^b,c^	3.76 ^a^	3.51 ^b,c^	3.65 ^b^
ANOVA														
Y	**	**	**	**	**	**	**	**	**	**	**	**	**	**
N	ns	ns	ns	ns	ns	Ns	ns	*	*	*	*	*	*	*
Y + N	ns	ns	ns	ns	ns	Ns	ns	ns	ns	ns	Ns	ns	ns	ns

Note: There is a column for each nitrogen rate; means followed by different lowercase letters are significantly different according to Tukey’s HSD test (0.05). Within a column, uppercase letters indicate comparisons among two nitrogen rates. * and **: significant at 0.01 and 0.05 probability levels, respectively; ns, not significant at 0.05 probability level.

**Table 2:** Effects of nitrogen rate on total nitrogen accumulation of different growth stages in dryland winter wheat.

Year	Anthesis Stage (AS)							Maturity Stage (MS)						
	N0	N90	N120	N150	N180	N210	N240	N0	N90	N120	N150	N180	N210	N240
2013–2014	60.10 ^f^	70.42 ^e^	81.70 ^d^	88.30 ^c^	97.80 ^a^	96.38 ^a^	93.46 ^b^	84.90 ^f^	113.30 ^e^	127.20 ^d^	142.92 ^b^	137.12 ^c^	148.30 ^a^	143.65 ^b^
2014–2015	86.50 ^g^	100.50 ^f^	122.20 ^e^	133.84 ^d^	152.82 ^c^	170.90 ^a^	166.74 ^b^	118.30 ^f^	162.40 ^e^	176.50 ^c^	173.22 ^d^	192.50 ^b^	199.86 ^a^	199.44 ^a^
2015–2016	90.92 ^f^	109.50 ^e^	118.40 ^d^	132.19 ^c^	138.80 ^b^	158.93 ^a^	155.21 ^a^	126.42 ^f^	147.20 ^e^	167.30 ^d^	175.89 ^c^	183.98 ^b^	188.20 ^a^	184.40 ^b^
2016–2017	77.84 ^d^	84.30 ^c^	88.20 ^c^	100.92 ^b^	103.30 ^b^	116.47 ^a^	114.18 ^a^	95.90 ^e^	120.40 ^d^	130.40 ^c^	148.98 ^ab^	146.98 ^b^	150.90 ^a^	143.34 ^b^
2017–2018	89.71 ^d^	98.81 ^c^	102.40 ^c^	104.60 ^c^	128.19 ^b^	144.47 ^a^	139.40 ^a^	104.60 ^e^	144.30 ^d^	158.80 ^c^	167.89 ^b^	176.55 ^ab^	178.50 ^a^	180.30 ^a^
2018–2019	88.21 ^f^	108.21 ^e^	117.80 ^d^	130.40 ^c^	144.80 ^b^	159.36 ^a^	157.92 ^a^	115.94 ^e^	155.90 ^d^	174.80 ^c^	185.74 ^b^	195.30 ^a^	198.32 ^a^	194.44 ^a^
2019–2020	63.32 ^e^	71.60 ^d^	79.40 ^c^	88.56 ^b^	90.40 ^b^	101.42 ^a^	95.56 ^b^	85.69 ^e^	107.34 ^d^	123.20 ^c^	144.72 ^a^	135.77 ^b^	145.89 ^a^	144.93 ^a^
2020–2021	73.94 ^e^	93.30 ^d^	98.90 ^c^	104.18 ^b^	106.27 ^b^	115.92 ^a^	120.34 ^a^	99.90 ^e^	128.30 ^d^	142.40 ^c^	150.92 ^a^	154.45 ^a^	149.89 ^b^	141.70 ^c^
ANOVA														
Y	*	*	*	*	*	*	*	*	*	*	*	*	*	*
N	**	**	**	**	**	**	**	**	**	**	**	**	**	**
Y + N	*	*	*	*	*	*	*	*	*	*	*	*	*	*

Note: There is a column for each nitrogen rate; means followed by different lowercase letters are significantly different according to Tukey’s HSD test (0.05). * and **, significant at 0.01 and 0.05 probability levels, respectively.

**Table 3:** Effects of nitrogen rate on nitrogen-use efficiency index in dryland winter wheat.

Year	Nitrogen-Use Efficiency (kg kg^−1^)							Nitrogen Partial Factor Productivity						
	N0	N90	N120	N150	N180	N210	N240	N0	N90	N120	N150	N180	N210	N240
2013–2014	-	11.12 ^b^	12.50 ^a^	12.66 ^a^	9.96 ^c^	8.30 ^d^	7.16 ^e^	-	36.72 ^a^	31.49 ^b^	28.0 ^c^	22.70 ^d^	19.23 ^e^	16.70 ^f^
2014–2015	-	13.62 ^b^	15.44 ^a^	12.10 ^c^	11.42 ^d^	8.65 ^e^	7.24 ^f^	-	52.64 ^a^	44.63 ^b^	35.44 ^c^	30.90 ^d^	25.30 ^e^	21.81 ^f^
2015–2016	-	8.14 ^e^	13.98 ^a^	11.66 ^b^	10.30 ^c^	9.70 ^d^	7.74 ^f^	-	47.55 ^a^	41.62 ^b^	35.86 ^c^	30.76 ^d^	26.66 ^e^	22.64 ^f^
2016–2017	-	9.11 ^c^	11.84 ^b^	11.50 ^a^	9.10 ^c^	7.10 ^d^	6.87 ^e^	-	36.42 ^a^	31.43 ^b^	29.44 ^c^	22.39 ^d^	18.96 ^e^	16.38 ^f^
2017–2018	-	14.84 ^b^	16.62 ^a^	13.82 ^b^	11.80 ^c^	10.79 ^d^	8.26 ^e^	-	46.86 ^a^	39.74 ^b^	34.34 ^c^	27.93 ^d^	23.54 ^e^	20.28 ^f^
2018–2019	-	13.49 ^b^	17.03 ^a^	13.16 ^b^	12.10 ^c^	10.10 ^d^	7.83 ^e^	-	48.82 ^a^	43.60 ^b^	35.65 ^c^	29.84 ^d^	24.28 ^e^	21.19 ^f^
2019–2020	-	8.24 ^c^	12.83 ^b^	13.36 ^a^	11.02 ^b,c^	9.82 ^c^	7.54 ^d^	-	34.25 ^a^	32.40 ^b^	29.93 ^c^	24.54 ^d^	19.93 ^e^	17.26 ^f^
2020–2021	-	10.23 ^c^	12.10 ^b^	11.44 ^a^	9.52 ^d^	8.64 ^e^	5.93 ^f^	-	41.82 ^a^	35.92 ^b^	31.76 ^c^	25.36 ^d^	21.17 ^e^	17.75 ^f^
Mean	-	11.01 ^b^	13.73 ^a^	12.23 ^a^	10.66 ^b^	9.73 ^c^	7.25 ^d^	-	43.13 ^a^	37.84 ^b^	32.76 ^c^	26.72 ^d^	22.53 ^e^	19.43 ^f^
ANOVA														
Y		*	*	*	*	*	*		*	*	*	*	*	*
N		**	**	**	**	**	**		**	**	**	**	**	**
Y + N		*	*	*	*	*	*		*	*	*	*	*	*

Note: Different lowercase letters within a column and different capital letters within a row or column represent significant differences (*p* < 0.05). Means and standard errors of three replicates are presented. The means are not significantly different within a given season when followed by the same lowercase letter using LSD at *p* < 0.05. “*” and “**”, indicate significant difference between the two optimal N rates in dry and normal year at 0.01 and 0.05 probability levels, respectively.

**Table 4:** Correlation analysis of nitrogen accumulation and yield and its components at different growth stages in dryland wheat.

Index	Grain Yield	Spike Number	Grain Number	Thousand Grain Weight
Sowing-Jointing	0.8612 **	0.8625 **	0.4488	0.6366
Jointing-Anthesis	0.9356 **	0.7925 *	0.7256 *	0.9156 **
Anthesis-Maturity	0.8322 **	0.6884	0.7324 *	0.7956 *

* and **: significant at 0.01 and 0.05 probability levels.

**Table 5:** Correlation analysis of LAI and yield and its components at different growth stages in dryland wheat.

Index	Grain Yield	Spike Number	Grain Number	Thousand Grain Weight
Nitrogen partial factor productivity	0.8156 *	0.9566 **	0.3546	0.4566
LAI of jointing	0.7445 *	0.8125 **	0.7006 *	0.3588
LAI of anthesis	0.8135 *	0.7239 **	0.8433 *	0.8156 **

* and **: significant at 0.01 and 0.05 probability levels.

**Table 6:** Basic nutrient contents in soil layer of 0–20 cm. Soil basic fertility of the test sites in 2013–2021 in dryland Wheat Loess Plateau.

Year	Organic Matter (g kg^−1^)	Total Nitrogen (g kg^−1^)	Alkaline Hydrolysis Nitrogen (mg kg^−1^)	Available Phosphorus (mg kg^−1^)
2013–2014	10.18	0.70	39.32	16.62
2014–2015	10.55	0.68	37.65	17.64
2015–2016	11.16	0.67	32.79	15.67
2016–2017	10.62	0.69	38.22	15.28
2017–2018	9.17	0.71	34.53	20.29
2018–2019	9.72	0.60	32.74	14.73
2019–2020	10.38	0.68	39.88	13.88
2020–2021	9.99	0.70	34.34	18.44

## Data Availability

Not applicable.

## Supplementary Materials

The following supporting information can be downloaded at: https://www.mdpi.com/article/10.3390/plants12122369/s1, Table S1. Effects of nitrogen rate on dry-matter accumulation at different growth stages in dryland winter wheat. Values are means ± SD, n = 3. Different lowercase letters indicate significant differences at *p* < 0.05. Table S2. Effects of nitrogen rate on translocation of pre-/post-anthesis accumulated dry matter to grain in dryland wheat. Values are means ± SD, n = 3. Different lowercase letters indicate significant differences at *p* < 0.05.
