# Supplementary material for: Long-Term Nutrient Cycle in Improved Grain Yield of Dryland Winter Wheat (Triticum aestivum L.) under Hydrological Process of Plant Ecosystem Distribution in the Loess Plateau of China
Source: Plants (Basel). 2023 Jun 19;12(12):2369. doi: 10.3390/plants12122369 (PMC10303035; doi:10.3390/plants12122369)
Supplement: Supplementary file 1 [file plants-12-02369-s001.zip › plants-2430800-supplementary.pdf]

plants

Table S1. Effects of Nitrogen rate on dry matter accumulation different grow stages in dryland winter wheat.

Year	Jointing stage (JS)							Anthesis stage (AS)							Maturity stage (MS)						
	N0	N90	N120	N150	N180	N210	N240	N0	N90	N120	N150	N180	N210	N240	N0	N90	N120	N150	N180	N210	N240
2013-2014	2.61 cd	2.62cd	2.86 bc	2.95b	3.24a	3.30a	3.24a	5.97ef	5.62d	6.20c	6..31b	6.93a	6.47bc	4.80e	5.80e	7.12d	8.40b	9.90a	9.50b	8.80bc	7.90c
2014-2015	2.52 d	2.64 de	3.12cd	3.35bc	3.45b	3.70a	3.61a	7.50d	8.10c	9.31b	9.77b	11.12a	9.60ab	9.80b	8.12d	11.27c	12.70b	12.78b	14.60a	12.48b	12.72b
2015-2016	3.09d	3.30 c	3.60c	3.54bc	3.81b	4.24a	4.38a	8.28d	8.80c	9.31b	10.33ab	10.64a	10.20a	10.99a	9.46d	10.55c	11.40b	11.97ab	12.20ab	12.70a	12.34ab
2016-2017	2.50 c	2.70bc	2.73bc	2.90b	2.86bc	2.94a	2.46c	5.87d	5.50c	6.10b	6.77a	6.30ab	6.18b	4.70d	6.10e	7.60d	8.64c	9.48a	9.10b	8.30c	7.50d
2017-2018	2.86 c	2.87c	2.95cd	2.80c	3.42b	3.57a	3.50a	7.49cd	7.45b	7.67b	7.24b	8.60ab	8.50ab	9.20a	7.50e	10.11d	11.21c	10.59cd	12.21a	11.60b	12.70a
2018-2019	2.28 f	2.47ef	2.72de	2.100c	3.11c	3.36ab	3.64a	7.60ef	7.77d	8.28c	9.11b	9.56a	8.30c	8.60c	7.74e	10.37d	11.81c	13.69b	15.60a	13.90b	14.45ab
2019-2020	2.77 ef	2.90d	2.98cd	3.18bc	3.51a	3.58a	3.67a	5.89d	5.99c	6.15c	6.72a	6.50b	5.97c	4.90d	6.10e	7.77d	9.20b	10.30a	10.11b	9.30b	8.75c
2020-2021	3.38 cd	3.56bc	3.70b	3.56b	3.63b	3.94a	3.96a	6.77d	7.57b	7.78b	8.20a	8.46a	7.60b	6.20c	7.30e	9.40c	10.14c	11.80a	11.64a	11.50ab	9.70d
ANOVA																					
Y	*	*	*	*	*	*	*	*	*	*	*	*	*	*	*	*	*	*	*	*	*
N	**	**	**	**	**	**	**	**	**	**	**	**	**	**	*	*	*	*	*	*	*
Y*N	*	*	*	*	*	*	*	*	*	*	*	*	*	*	*	*	*	*	*	*	*

Note: The column for each Nitrogen rate, means followed by different lower-case letters are significantly different according to Tukey's HSD test (0.05). Within a column, upper-case letters indicate comparisons among two Nitrogen rate. * and **, significant at 0.01 and 0.05 probability levels, respectively; ns, not significant at 0.05 probability level.

Table S2. Effects of Nitrogen rate on translocation of Pre-/Post anthesis accumulated dry matter to grain in dryland wheat.

Year	Translocation of Pre-anthesis accumulated dry matter to grain							Translocation of Post-anthesis accumulated dry matter to grain						
	N0	N90	N120	N150	N180	N210	N240	N0	N90	N120	N150	N180	N210	N240
2013-2014	1.50 e	1.94 b	1.76 c	1.60 d	1.70 d	1.94 b	2.25 a	0.90 g	1.51 f	2.20 d	2.82 a	2.65 b	2.34 c	2.05 e
2014-2015	1.93 e	1.62 f	2.15 d	2.66 b	2.26 c	2.75 a	2.69 b	1.66 e	3.26 c	3.40 bc	3.46 b	3.56 a	2.89 d	2.95 d
2015-2016	1.41 f	1.66 e	2.56 b	2.90 a	2.10 d	2.05 d	2.41 c	2.22 d	2.78 a	2.75 a	2.68 b	2.78 a	2.73 a	2.30 c
2016-2017	1.31d	1.31 d	1.55 b	1.57 b	1.57b	2.11 a	1.46 c	1.24 f	2.10 e	2.52 d	2.72 b	2.60 c	2.09 e	2.92 a
2017-2018	1.87 b	1.70 c	1.54 d	1.90 b	1.64 c	2.16 a	1.89 b	1.08 e	2.68 a	3.54 b	3.37 c	3.60 ab	3.08 d	3.16 cd
2018-2019	1.36 b	1.72 a	1.66 b	1.70 a	1.64 b	1.74 a	1.72 a	1.69 e	2.63 d	3.56 c	3.68 b	3.82 a	3.65 b	3.56 bc
2019-2020	1.22d	1.41 c	1.37 c	1.57 b	1.78 a	1.84 a	1.61 b	1.18 e	1.79 d	3.09 c	3.62 a	3.63 a	3.27 b	3.25 b
2020-2021	1.33 e	2.09 a	2.10 a	1.67 d	1.70 d	1.88 b	1.74 c	1.58 g	1.86 f	2.40 e	3.36 a	3.14 c	3.29 b	3.04 d
Mean	1.49 f	1.81 e	1.94 d	2.08 c	2.11 c	2.30 a	2.22 b	1.45d	2.33 c	2.94 b	3.21 a	3.22 a	2.93 b	2.91 b
ANOVA														
Y	*	*	*	*	*	*	*	*	*	*	*	*	*	*
N	**	**	**	**	**	**	**	**	**	**	**	**	**	**
Y*N	ns	ns	ns	ns	ns	ns	ns	Ns	Ns	Ns	Ns	Ns	Ns	Ns

Note: Translocation of Pre-anthesis accumulated dry matter to grain: Translocation of Post-anthesis accumulated dry matter to grain. The column for each Nitrogen rate, means followed by different lower-case letters are significantly different according to Tukey's HSD test (0.05). Within a column, upper-case letters indicate comparisons among two Nitrogen rate. * and **, significant at 0.01 and 0.05 probability levels, respectively; ns, not significant at 0.05 probability level.
